# Sustainable treatment success of an Os naviculare syndrome using conservative measures, infiltration therapy, and shock waves

**DOI:** 10.1093/jscr/rjae544

**Published:** 2024-08-28

**Authors:** Julian Ramin Andresen, Sebastian Radmer, Stephan E Puchner

**Affiliations:** Department of Orthopedics and Trauma Surgery, Medical University of Vienna, Vienna, Austria; Specialist Practice for Orthopedics, Centre for Orthopedics, Berlin, Germany; Department of Orthopedics and Trauma Surgery, Medical University of Vienna, Vienna, Austria

**Keywords:** accessory tarsal bones, immobilizing foot pain, os tibiale externum, os naviculare accessorium, os naviculare syndrome, shockwave therapy

## Abstract

Medial plantar foot pain can have various causes, and the painful Os tibiale externum should be considered in the differential diagnosis. A reliable diagnosis can be made through physical examination and multimodal imaging. We report on a 53-year-old man with severe, load-dependent pain consistent with an accessory navicular syndrome, caused by a pes planovalgus, which consecutively induced focal inflammation and tenosynovitis of the tibialis posterior tendon. Multifactorial conservative measures, including infiltration therapy, provided only moderate symptom relief. A final shockwave therapy ultimately led to a sustainable symptom relief.

## Introduction

The Os tibiale externum (synonyms: Os naviculare accessorium, Os naviculare externum, Os naviculare secundarium, prehallux) is an accessory ossicle located dorsomedially to the plantar-oriented tuberosity of the navicular bone [1]. It is one of the most common ossicles in the human foot skeleton, along with the Os trigonum and the Os peroneum, with prevalence rates varying significantly across different publications [2–5]. The prevalence of the Os tibiale externum in adults ranges from 4% to 23.3%, with bilateral occurrence in over 50% of cases, and women being more frequently affected than men [6–8]. Conversely, Lee *et al*. [3] found a prevalence of 34% in a study of 448 healthy Koreans. The Os tibiale externum was first described by Bauhin in 1605 [9]. According to Geist [9] and a revision by Lawson *et al*. [10], three types of Os tibiale externum are distinguished. Type I is characterized by a 2–3-mm oval ossicle, slightly separated from the navicular bone, acting as a sesamoid bone in the distal part of the tibialis posterior tendon. Type II is triangular with a diameter of up to 12 mm. It is considered a secondary ossification center in the cartilaginous structure of the navicular bone, connected to the tuberosity of the navicular bone by fibrous cartilage. Portions of the tibialis posterior tendon insert atypically into the Os tibiale externum. Type III is a synostosed Type II ossicle, appearing as a prominent tuberosity, the Os naviculare cornutum. Alsager *et al*. [8] found a distribution of 41.2% for Type I, 33.5% for Type II (combined with 13.9% for Type IIa and 19.6% for Type IIb), and 24.7% for Type III in a study of 117 patients with an accessory navicular bone. Huang *et al*. [11] reported 41.6% for Type I and Kalbouneh *et al*. [7] reported 25.4%, while Type II was found in 36.8% and 42.4%, and Type III in 21.6% and 32.0%, respectively. The wide variation in prevalence rates of the Os tibiale externum and its subtypes is influenced by different populations concerning race, age, gender, measurement methods, and data collection questions [3]. In summary, the Os tibiale externum is not uncommon and must be considered in radiological diagnostics, clinical examination, and critical assessment of findings [6].

We report on a patient with a Type II Os tibiale externum presenting with subacute to chronic medioplantar foot pain.

## Case report

**Figure 1:**
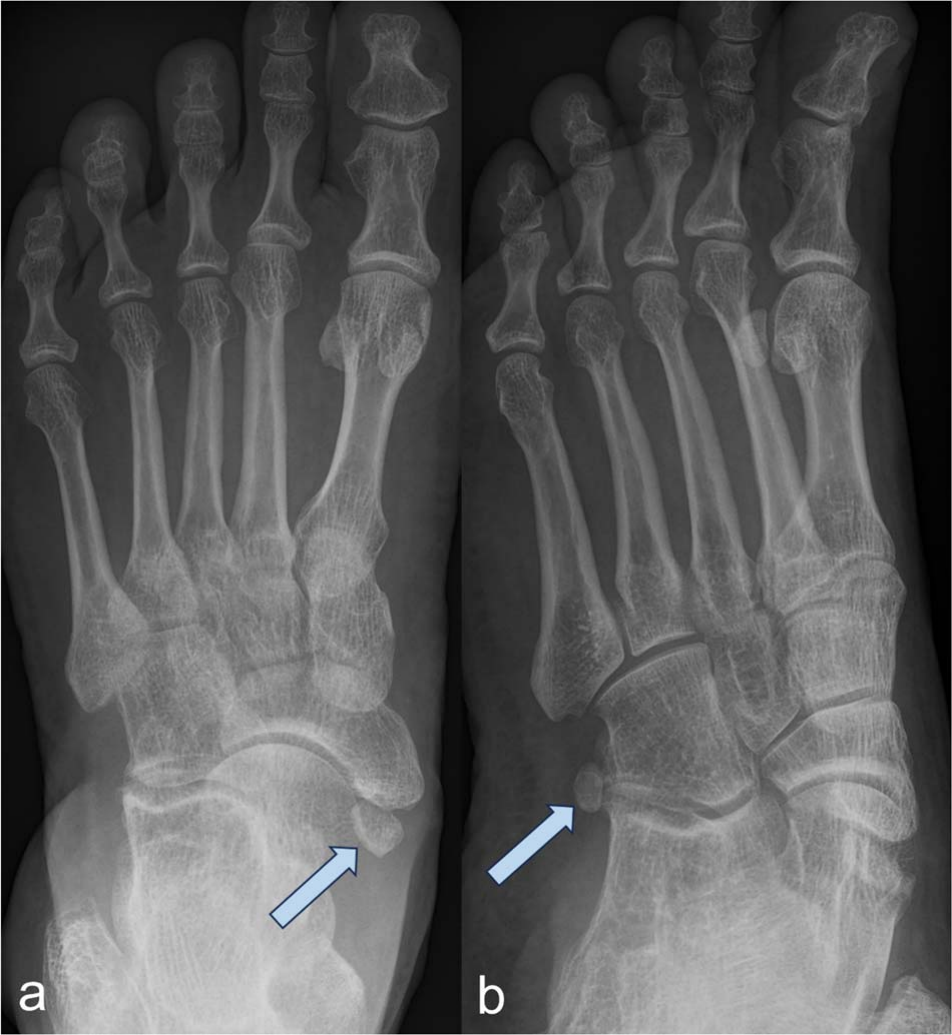
Image of an Os tibiale externum Type II in the anterior–posterior (a) and oblique (b) radiographic view; the ossicle is marked with an arrow.

**Figure 2:**
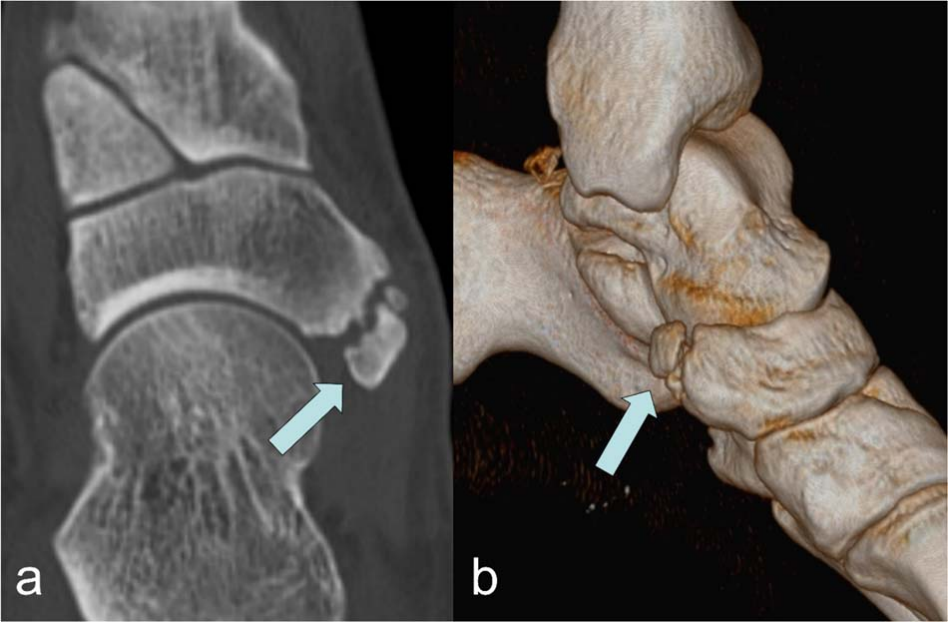
Image of the Os tibiale externum Type II in the coronal reformatted CT scan (a) and in the 3D representation (b); the ossicle is marked with an arrow.

**Figure 3:**
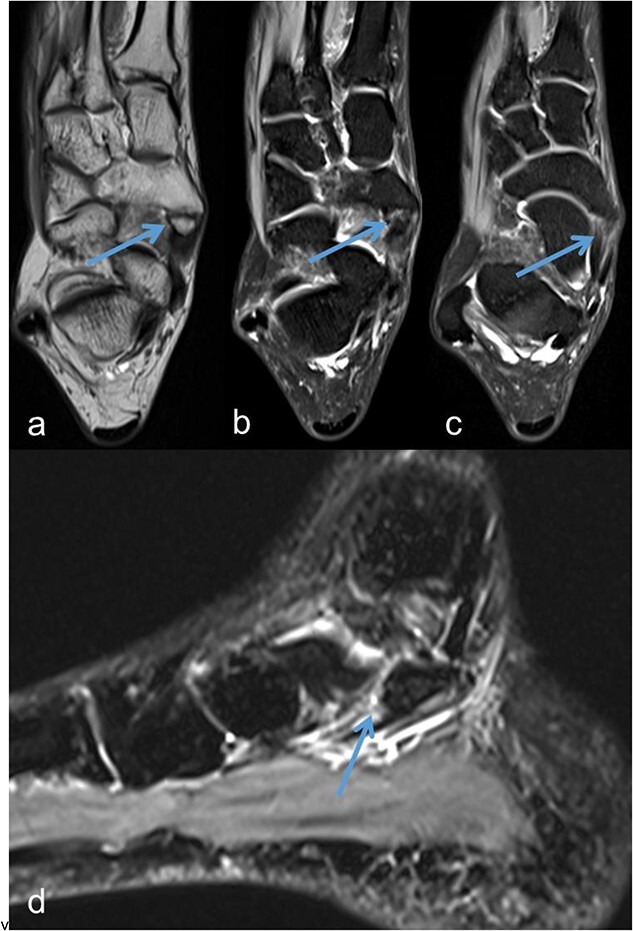
Os tibiale externum Type II in the coronal T1-weighted MRI scan (a), inflammatory reaction/tendinosis at the insertion of the tendon of the tibialis posterior muscle visualized in the heavily T2-weighted, fat-suppressed MRI scans (b and c), synchondrosis between the Os tibiale externum Type II and Os naviculare with central and perifocal edema visualized in the sagittal heavily T2-weighted, fat-suppressed MRI scan (d); each marked with an arrow.

A 53-year-old man with a body mass index of 22.3 kg/m^2^ reported severe, load-dependent pain (6 out of 10 on the VAS) in the left foot, which had increased over the past two months. Light jogging as a recreational sport was no longer possible due to pain, and no trauma was recalled. Clinically, there was a mild pes planovalgus, with slight swelling and warmth medioplantarly and distal to the medial malleolus along the course of the tibialis posterior tendon. A marked point tenderness was noted, and forced dorsiflexion was severely painful. Conventional radiographs showed a triangular Type II Os tibiale externum at the typical site, measuring approximately 10.5 x 6 mm (Fig. 1a and b). Additional CT imaging in the axial plane with coronal reconstruction and subsequent 3D reconstruction (Fig. 2a and b) revealed a Type II Os tibiale externum with a distinct synchondrosis gap to the navicular bone. An MRI showed central inflammatory reaction in the synchondrosis and significant perifocal soft tissue edema of the Os tibiale externum with involvement of the tibialis posterior tendon, with no tendon rupture detected (Fig. 3a–d).

Therapeutically, the foot was relieved and immobilized with a lower leg-foot orthosis (Vacoped-Orthosis®) for 6 weeks. Supportive analgesic/anti-inflammatory systemic therapy with a nonsteroidal antirheumatic drug (Etoricoxib 90 mg, Etoricoxib-ratiopharm®) was administered once daily for 14 days. The patient was also instructed to apply cryotherapy several times a day as directed.

With no significant clinical improvement, an additional ultrasound-guided infiltration therapy (injection of 2 ml of a drug mixture consisting of 1 ml of 2.5 mg dexamethasone (Lipotalon®) and 1 ml of bupivacaine (Carbostesin® 0.5%) with three repetitions at 7-day intervals) was performed, leading to a slight reduction in symptoms.

**Figure 4:**
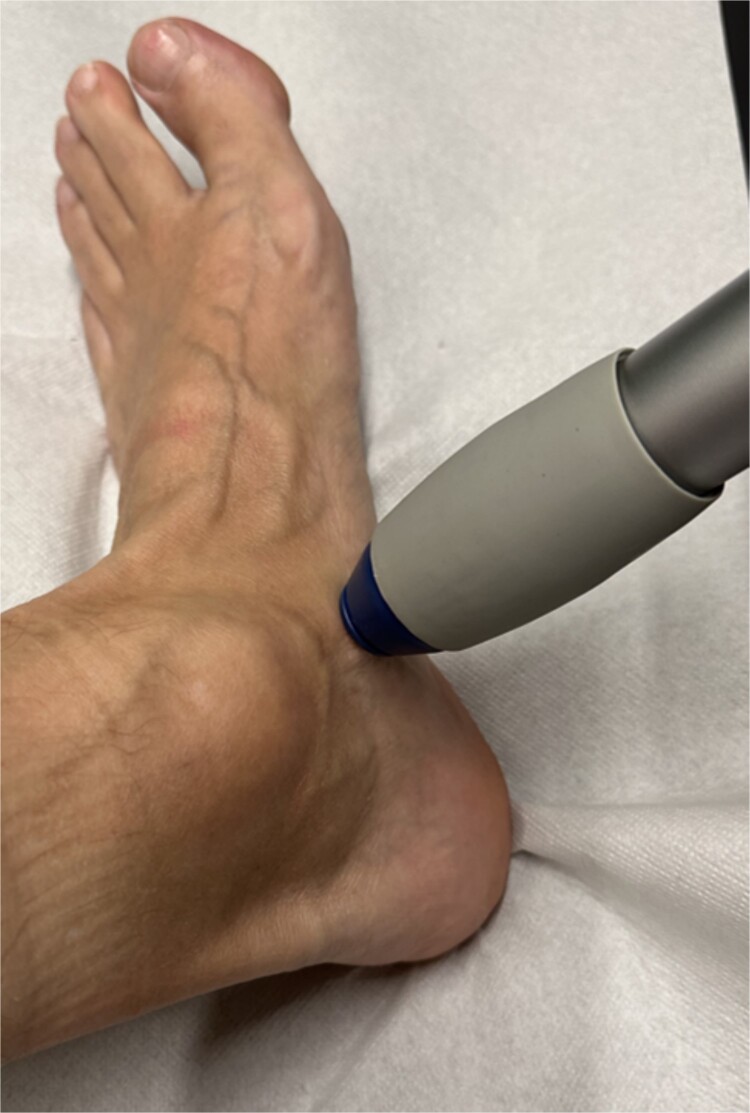
Illustration of the technical implementation of extracorporeal shockwave therapy.

Subsequently, five sessions of extracorporeal radial shock wave therapy at 3-day intervals, each with 4000 impulses, 1 bar, 20 Hz (STORZ MEDICAL, Duolith SD1) were administered (Fig. 4), resulting in significant and sustained pain relief.

After removal of the orthosis, physiotherapeutic treatment with manual therapy and physical therapy was initiated, and insoles were fitted to correct the existing foot deformity.

After the completion of therapeutic measures, 9 weeks after the initial presentation, the patient was pain free, and the foot was fully loadable. Recreational sports could be resumed without any problems. Over a follow-up period of an additional 9 months, no recurrence occurred.

## Discussion

Most cases of incidentally diagnosed Os tibiale externum are asymptomatic, with fewer than 1% showing painful symptoms [7]. However, with painful movement restriction and increasing pain at the medioplantar edge of the foot under palpation behind the navicular bone and during dorsiflexion, along with focal warmth, an Os naviculare syndrome should be considered [12]. A symptomatic Os tibiale externum is often associated with flatfoot deformity, as in our case [13]. The flattening of the longitudinal arch can result in increased stress at the insertion of the tibialis posterior tendon and the synchondrosis between the Os tibiale externum and the navicular bone, leading to perifocal inflammation with edema formation and tendinosis/tendovaginitis. This process is painful and aggravates under load and external pressure [14]. In the presence of an Os tibiale externum on conventional radiographs and corresponding clinical findings, MRI, especially with strongly T2-weighted, fat-suppressed sequences, can visualize the extent of perifocal, osseous, and cartilaginous edema and inflammation or a beginning rupture of the tibialis posterior tendon [15, 16]. The extent of bony remodeling can also be assessed by quantitative single-photon emission computed tomography/computed tomography (SPECT/CT) [17]. The results of these examinations influence the therapeutic options, with conservative management including immobilization, analgesic/anti-inflammatory medication with a nonsteroidal antirheumatic drug, steroid injection, and individually fitted shoe inserts and shoe forms being the primary approach [18, 19]. In a study by Wynn *et al*. [19], among 169 adolescent patients with symptomatic Os tibiale externum, conservative measures led to complete pain relief in only 28%, partial pain reduction with tolerable clinical symptoms in 41%, and 30% were referred for surgery.

Although not age-comparable, conservative measures, including steroid infiltration, did not yield satisfactory results in our patient. Subsequently, shock wave therapy led to significant and sustained pain relief. Radial shock waves spread low-energy waves diffusely, stimulating cytokine production in the cells, increasing cellular metabolism, and improving tissue perfusion in the treated region, thereby promoting the healing process. The application is usually mildly painful. Shock wave therapy is an effective and safe, noninvasive procedure in the treatment of tendinopathies [20], with the most experience in the foot area being in the treatment of chronic plantar fasciitis with good results [21]. There are two case reports of targeted use in painful Os peroneum syndrome [22, 23], but to our knowledge, no reports exist on shock wave treatment for Os tibiale externum syndrome.

Surgical tendon reconstruction was not indicated in our case, as MRI did not reveal any tearing or rupture of the tibialis posterior tendon. If no improvement had been observed after the shock wave treatment, we would have advised the patient to undergo surgical intervention, as surgical procedures either through isolated excision of the ossicle or with tendon reconstruction according to Kidner show good results with a low complication rate [24].

## Conclusion

In cases of medioplantar foot pain and the presence of a Type II Os tibiale externum, an accessory navicular syndrome should be considered in the differential diagnosis. A flattening of the foot arch can be the underlying cause, which can be corrected with customized shoe inserts. Inflammatory reactions in the region of the Os tibiale externum and the tibialis posterior tendon can be managed with immobilization, antiinflammatory medication, steroid infiltration, and shock wave therapy. In our case, complete and sustained symptom relief was achieved only after shock wave therapy. In the therapeutic cascade for treating symptomatic Os tibiale externum, the option of shock wave therapy should be considered before surgical intervention, provided there is no rupture of the tibialis posterior tendon.
